# Safety and Analgesic Consumption Impact of Continuous Peripheral Regional Analgesia in Earthquake-Related Crush Injury: A Retrospective Cohort Study

**DOI:** 10.3390/jcm15218263

**Published:** 2026-10-23

**Authors:** Özal Adıyeke, Ergun Mendes

**Affiliations:** 1 Department of Anesthesiology and Reanimation, Acıbadem Maslak Hospital, 34457 Istanbul, Turkey; 2 Department of Anesthesiology and Reanimation, Başakşehir Çam ve Sakura City Hospital, University of Health Sciences, 34480 Istanbul, Turkey; 3 Department of Anesthesiology and Reanimation, Koç University Faculty of Medicine Hospital, 34010 Istanbul, Turkey; erg.mendes@gmail.com

**Keywords:** crush syndrome, earthquake, disaster medicine, regional anesthesia, peripheral nerve catheter, acute kidney injury, opioid consumption, morphine milligram equivalents, compartment syndrome, propensity score

## Abstract

**Background**: The 6 February 2023 Kahramanmaraş earthquake caused an unprecedented burden of crush-related extremity trauma. Continuous peripheral regional analgesia is frequently used in these patients, but its safety regarding renal outcomes and its effect on systemic opioid consumption have not been quantified in comparative cohort data. **Methods**: We conducted a retrospective, exploratory cohort study of 181 hospitalizations (141 patients) following the earthquake, comparing patients who received a continuous peripheral nerve catheter with those managed without one. No prospective sample-size calculation was performed. The primary outcome was acute kidney injury (AKI), defined per adapted KDIGO criteria; the secondary outcome was systemic opioid exposure, assessed both as opioid-days and, in a dose-based sensitivity analysis, as morphine milligram equivalents (MME). Because patients contributed multiple admissions, regression models used cluster-robust standard errors. A propensity-score-matched sensitivity analysis (age, sex) addressed confounding by indication, and intensive care unit (ICU) days were added as a further covariate in sensitivity models. **Results**: Catheter and non-catheter groups had similar age (*p* = 0.369) but differed in length of stay (median 42 vs. 16 days) and surgical procedures (10 vs. 2), reflecting catheter-selection criteria; detailed injury-severity and comorbidity data were not available for either group. Catheter use was not significantly associated with AKI (adjusted OR 0.49, 95% CI 0.16–1.47, *p* = 0.203) or with systemic opioid-day rate (adjusted RR 1.15, 95% CI 0.88–1.51, *p* = 0.294). Results were materially unchanged after adding ICU days as a covariate (AKI OR 0.42, *p* = 0.148, *n* = 123; opioid-days RR 1.23, *p* = 0.223, *n* = 160) and when opioid exposure was expressed as MME per day (adjusted ratio 1.32, 95% CI 0.73–2.38, *p* = 0.361, *n* = 165). A propensity-matched analysis (26 pairs) was concordant for all outcomes. **Conclusions**: In this exploratory, hypothesis-generating cohort, we did not detect a significant association between continuous regional analgesia and either AKI risk or opioid consumption across six complementary analyses. These non-significant findings do not establish equivalence or safety, particularly given wide confidence intervals, a modest number of events, incomplete clinical characterization, and residual confounding that could not be fully resolved with the available data; they should be regarded as hypothesis-generating pending confirmation in better-characterized, adequately powered, multicenter cohorts.

## 1. Introduction

The 6 February 2023 Kahramanmaraş earthquake sequence produced a mass-casualty burden of crush-related extremity trauma rarely documented in the anesthesiology literature [1,2], with crush syndrome—rhabdomyolysis, hyperkalemia, coagulopathy, and acute kidney injury (AKI) arising from ischemia–reperfusion injury [3,4]—reported in roughly one-third of orthopedic admissions and postoperative dialysis independently associated with mortality [2]. Iterative debridement and staged reconstruction generated a population requiring prolonged, repeated analgesia over weeks rather than days.

Continuous peripheral regional analgesia is an attractive strategy in this setting, offering dense analgesia across multiple surgical sessions while theoretically sparing systemic opioids [5]; peripheral nerve blockade is increasingly integrated into multiple-trauma care more broadly [6], including as primary analgesic management in orthopedic-surgical emergency settings [7]. Our group previously reported that it improved quality-of-recovery (QoR-15) [8] scores in a single-arm cohort of 26–29 catheterized earthquake victims [9], without a comparison group or objective safety endpoints. Two concerns temper enthusiasm for the technique in extremity trauma: dense blockade may mask the breakthrough pain that heralds acute compartment syndrome (ACS) [10,11], yet the evidence is limited to isolated case reports—two independent systematic reviews each identified only six such reports, with no comparative or cohort-level data, and both concluded that breakthrough pain through a functioning catheter remained a reliable warning sign in most reported cases [12,13,14]; and prolonged catheter dwell time, an inevitability in a repeated-debridement program, is a recognized risk factor for perineural catheter infection [15,16,17]. A further, complementary rationale is worth stating explicitly: because most patients in this population undergo fasciotomy at their index surgical treatment, the acute diagnostic concern that a catheter might mask evolving ACS applies most directly to that first procedure, whereas for the serial debridements that follow, a well-functioning catheter’s principal value may lie in permitting repeated regional, rather than general, anesthesia, reducing cumulative general-anesthetic exposure across multiple trips to the operating room—a distinct, clinically important potential benefit that the present study was not designed to test (Section 4.5).

No study has examined whether the choice of analgesic technique itself is associated with renal outcomes or systemic opioid exposure in this population—a question within the anesthesiologist’s area of responsibility, and one that must take seriously both confounding by indication [18] (catheter allocation was clinician-directed, not randomized) and the statistical consequences of patients contributing multiple, non-independent hospitalizations [19,20]. We therefore performed an exploratory retrospective cohort study integrating medication-order, laboratory, transfusion and surgical records to test whether continuous catheter use was associated with (i) AKI and (ii) systemic opioid consumption, using cluster-robust regression, correctly specified count models, dose-based (MME) and ICU-adjusted sensitivity analyses, and propensity-score matching. Mass-casualty disaster cohorts of this kind rarely permit randomized or even closely matched designs, given that treatment decisions are made under triage conditions rather than research protocols; our aim here is accordingly not to establish causal efficacy, but to document, as transparently and rigorously as the available data allow, the real-world pattern of renal and analgesic outcomes associated with a widely used anesthesia technique in this setting. We present this explicitly as hypothesis-generating: the available institutional dataset does not include a formal injury-severity index, comorbidity profile, or amputation status, and no prospective power calculation was performed, limitations we return to in Section 4.5. The adjusted and sensitivity analyses that follow are accordingly best understood not as establishing a causal effect, but as a transparent test of how robust the raw, unadjusted association is to successive, increasingly rigorous scrutiny.

## 2. Methods

### 2.1. Design and Setting

This was a retrospective, single-center, exploratory cohort study conducted at SBÜ Başakşehir Çam ve Sakura City Hospital, Istanbul, Türkiye, a tertiary referral center that received a large volume of casualties transferred from the earthquake-affected region beginning 6 February 2023. Reporting follows the STROBE recommendations for cohort studies [21] (see Supplementary Materials). The study was approved by the Çam and Sakura City Hospital Ethics Committee as a retrospective review of earthquake-victim patients (Decision No. 2023-194; date of approval: 10 May 2023).

### 2.2. Population

All adult patients admitted with earthquake-related trauma who had at least one recorded medication order, laboratory result, surgical record, or blood product transfusion in the institutional dataset were eligible for this exploratory cohort; no prospective sample-size or power calculation was performed, and the final sample reflects the complete available institutional dataset rather than a pre-specified target (Section 4.5). The final analytic cohort comprised 181 hospitalizations corresponding to 141 unique patients; 37 hospitalizations, in patients who received at least one continuous peripheral nerve catheter, constituted the catheter group, and 144 constituted the non-catheter (systemic analgesia only) group. Beyond age, sex, length of stay, and ICU/ward days, detailed characterization of the study population—comorbidities, baseline renal function, a formal injury-severity score, amputation status, and the anatomical extent of crush injury—was not available in the source institutional records; this is a central limitation of the present analysis and is discussed explicitly in Section 4.5. Patients with more than one hospitalization contributed one row per admission for admission-level variables (length of stay, laboratory trajectory, opioid-days), while person-level variables (catheter status, sex) were assigned once per person and are shared across that person’s admissions; the resulting within-patient correlation is addressed statistically in Section 2.4.

### 2.3. Data Sources and Variable Definitions

The primary outcome was acute kidney injury (AKI); the secondary outcome was systemic opioid exposure, assessed both as opioid-days and, in a dose-based sensitivity analysis, as morphine milligram equivalents (MME). Both are defined in full below, alongside the data sources from which they and the covariates were derived.

Data were extracted from four institutional sources covering the hospitalization course: (i) daily medication administration orders, from which antibiotic-days and narcotic-days were derived; (ii) daily biochemical and hematological laboratory panels, including age, sex, and intensive care unit (ICU) and ward (service)-day counts as recorded at admission and during the stay; (iii) blood and blood product transfusion records; and (iv) a surgical log identifying operative dates. Length of stay was defined as the number of distinct calendar days with a recorded medication order for that hospitalization. ICU days were recorded directly where available; where the ICU-day field was blank but the ward (service)-day field alone accounted for essentially the full length of stay, ICU days were inferred to be zero (verified by internal consistency against total length of stay); admissions with an explicitly ongoing ICU stay at the time of data extraction, or with neither field recorded, were treated as missing for this variable.

Systemic opioid exposure was assessed in two ways. First, as ‘narcotic-days’: the number of distinct hospitalization-days on which a systemic opioid analgesic was administered—tramadol (all formulary brand-equivalents), morphine, pethidine, or transdermal fentanyl—excluding non-opioid analgesics (paracetamol). Second, as a dose-based sensitivity analysis, the morphine milligram equivalent (MME) content of each order was estimated from the per-unit strength stated in the order text (e.g., tramadol 100 mg per ampoule/tablet) using standard equianalgesic conversion factors (tramadol ×0.1, morphine ×1.0, pethidine ×0.1), summed across all qualifying order lines per admission; oral tramadol drops (variable drop count, 39 order lines) and transdermal fentanyl (continuous-release dosing, 11 order lines) could not be reliably converted to a discrete per-order MME and were excluded from this specific calculation, representing under 1% of narcotic-related order lines. Catheter status was defined as receipt of a continuous peripheral nerve catheter at any point during the patient’s care. The anatomical site and specific type of catheter were not uniformly coded and could not be reported by block type; acute compartment syndrome diagnoses and fasciotomy-specific rates could not be reliably isolated from the surgical log; catheter-site infection was not separately coded and is not reported as an outcome (Section 4.4).

Acute kidney injury was defined as a rise in serum creatinine of ≥0.3 mg/dL or ≥1.5-fold between the first available and the peak in-hospital value, adapting the KDIGO serum-creatinine criterion [22]. A true pre-hospital baseline creatinine was unavailable for this disaster-triage population, so the first in-hospital value was used as a pragmatic reference; this will tend to underestimate AKI already present at admission (Section 4.5).

### 2.4. Statistical Analysis

Because several complementary analyses are used below, we summarize their purpose in plain terms before the technical detail: (a) a primary cluster-robust regression for each outcome (Section 3.2 and Section 3.3), adjusting for age and length of stay; (b) a sensitivity check adding ICU days as a further covariate (Section 3.4); (c) a sensitivity analysis replacing the opioid-days outcome with an estimated administered dose (MME/day, Section 3.4); and (d) a propensity-score-matched comparison as a non-regression-based check on confounding by indication (Section 3.5). All four reached the same qualitative conclusion for both outcomes, summarized in Section 3.5.

No prospective sample-size or power calculation was performed; this is an exploratory, hypothesis-generating analysis of the complete available institutional dataset, and the resulting event numbers—particularly 29 AKI events in the primary model—should be interpreted with this in mind. Continuous variables are reported as median (interquartile range) and compared between catheter groups using the Mann–Whitney U test. Because 141 patients contributed 181 hospitalizations, all regression-based comparisons used cluster-robust (sandwich) standard errors with the patient as the clustering unit [19,20].

**Figure 1:**
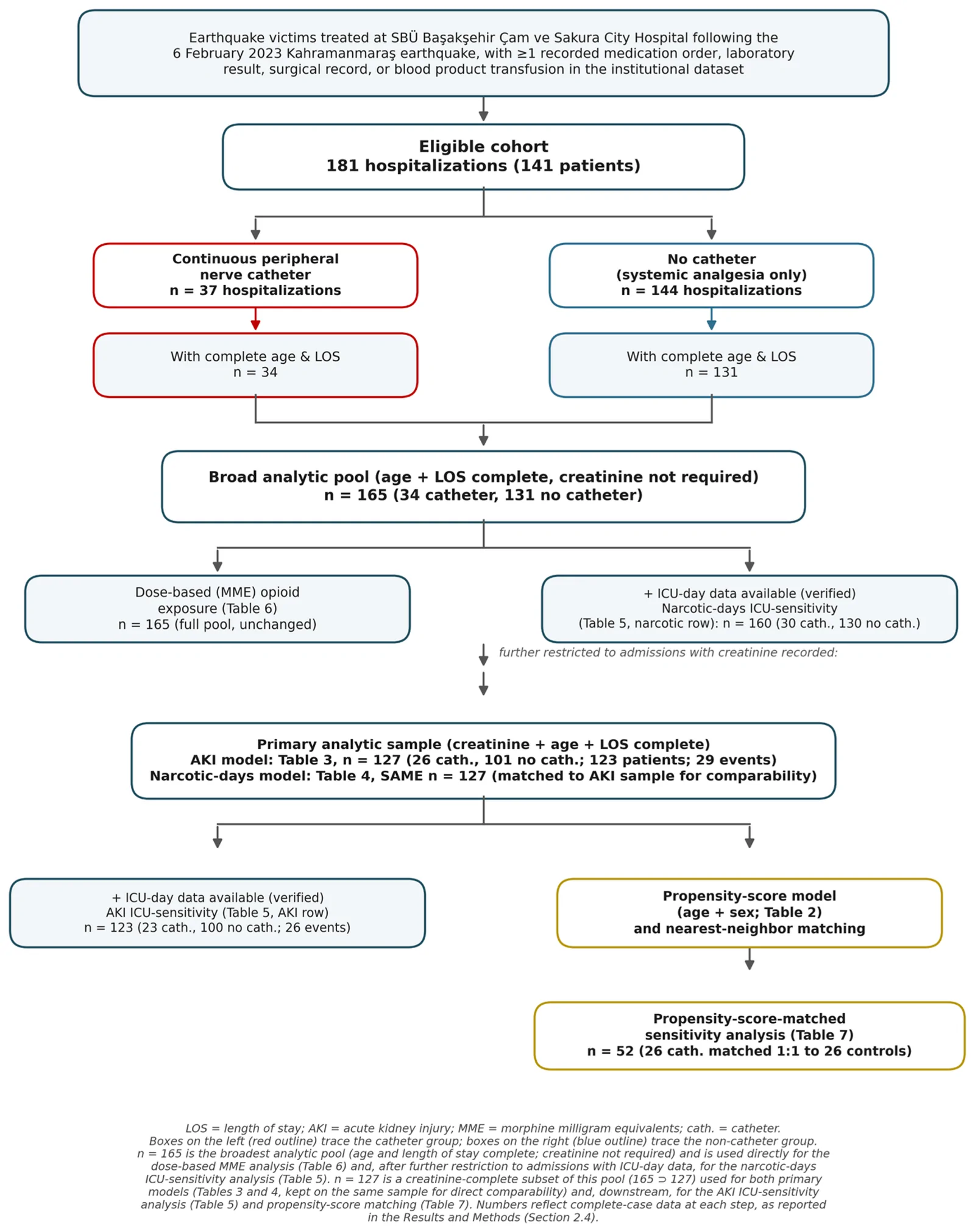
Patient flow diagram across the eligible cohort and the analytic subsamples used in Table 2, Table 3, Table 4, Table 5, Table 6 and Table 7.

The association between catheter status and AKI was estimated using multivariable logistic regression with cluster-robust standard errors, adjusted for age and log-transformed length of stay. The association between catheter status and opioid-days was estimated using maximum-likelihood negative binomial regression with log length of stay as an offset and cluster-robust standard errors, adjusted for age (reported in preference to a Poisson model, given overdispersion: Pearson χ^2^/df = 8.65). This primary narcotic-days model was estimated in the same creatinine-complete subsample used for the AKI model (*n* = 127) to keep the two primary analyses directly comparable; the broader age-and-length-of-stay-complete sample (*n* = 165), which does not require creatinine data, was used only for the dose-based (MME) and ICU-adjusted narcotic-days sensitivity analyses (Section 3.4), which are not constrained by the AKI outcome’s data requirements (Figure 1). Surgical procedure count was not included as an independent covariate in either model because of its high collinearity with length of stay (*r* = 0.75).

Two sensitivity analyses were added in response to peer review. First, ICU days (Section 2.3) were added as an additional covariate to both primary models, in the subset of admissions with non-missing ICU-day data. Second, the opioid-days outcome was supplemented with a dose-based analysis: total MME per admission was divided by length of stay to give an MME rate, log-transformed (ln[MME/day + 1]) and modeled by cluster-robust linear regression adjusted for age, directly paralleling the opioid-days model. Third, a propensity score for catheter receipt was estimated by logistic regression on the only reliably available pre-treatment covariates, age and sex [18,23], and catheter-treated patients were matched 1:1 to non-treated patients by greedy nearest-neighbor matching on the logit propensity score (caliper 0.2 SD), with primary outcome models re-estimated in the matched sample. Two-sided *p* < 0.05 was considered statistically significant throughout; given the exploratory nature of the study and the number of models fitted, *p*-values should be interpreted descriptively rather than as confirmatory hypothesis tests. Analyses were performed in Python 3.12.3 (pandas 3.0.2, scipy 1.17.1, and purpose-written iteratively reweighted least-squares implementations of logistic, negative binomial, and linear regression with cluster-robust sandwich variance estimation).

### 2.5. Use of Generative Artificial Intelligence

Generative artificial intelligence (GenAI) was used in the preparation of this study and manuscript. The tools were Claude Sonnet 4.6 (model identifier claude-sonnet-4-6) and Claude Sonnet 5.5 (claude-sonnet-5-5), both developed by Anthropic PBC (San Francisco, CA, USA) and used through Anthropic’s Claude application between July and October 2026. The tools were used (i) to consolidate, clean, and cross-match the four institutional data sources into the analytic dataset described in Section 2.2 and Section 2.3; (ii) to write and run the Python code for the statistical analyses described in Section 2.4; (iii) to identify the published literature cited; and (iv) to draft and edit the manuscript text. All data linkages, derived variables, analysis outputs, references, and text produced with these tools were reviewed, verified, and edited by the authors, who take full responsibility for the content of this publication. The tools are not authors and had no role in the interpretation of the findings or in the conclusions of the study.

## 3. Results

Results are organized as follows: Section 3.1 describes the propensity-score model used for later matching; Section 3.2 and Section 3.3 report the two primary, pre-specified analyses (AKI and opioid-days); and Section 3.4 and Section 3.5 report sensitivity analyses added in response to peer review (ICU-day adjustment, a dose-based opioid measure, and propensity-score matching), all of which reached conclusions consistent with the primary analyses (summarized in Section 3.5).

The cohort comprised 181 hospitalizations (141 patients; 37 catheter, 144 non-catheter). Figure 1 shows how this eligible cohort narrows across the several analytic subsamples used below. Table 1 summarizes the unadjusted comparison between groups; comorbidity, baseline renal function, injury-severity, and amputation data were not available for either group (Section 2.2).

Age was balanced between groups, but the catheter group had markedly longer length of stay and a substantially greater number of surgical procedures, consistent with the clinical practice of reserving continuous catheters for patients anticipated to require a repeated-debridement program; because no injury-severity measure was available, the extent to which this reflects true underlying injury severity versus treatment-pattern differences cannot be determined from these data (Section 4.5). The catheter group also had a numerically lower proportion of male patients than the non-catheter group (35% vs. 51%, among the 127 patients with complete covariate data); formal outcome stratification by sex was not performed; given the already modest catheter-group size (37 hospitalizations, 127 in the covariate-complete subset), sex-stratified subgroups would be too small to yield stable estimates, so sex was instead retained as a covariate in the propensity-score model (Table 2).

### 3.1. Propensity-Score Model and Matching

A logistic model for catheter receipt, using the only reliably available pre-treatment covariates (age and sex), is shown in Table 2.

**Table 2:** Propensity-score model for catheter receipt (*n* = 127).

Predictor	OR	95% CI	*p*
Age (per year)	1.00	0.97–1.02	0.945
Sex (male vs. female)	0.50	0.20–1.23	0.129

Matching produced 26 catheter-treated patients matched to 26 non-treated patients; age and sex were well balanced post-matching, but length of stay remained markedly different (median 58 vs. 17.5 days), indicating that age and sex alone could not capture the injury-severity dimension driving catheter selection (Section 4.5).

### 3.2. Acute Kidney Injury

The primary multivariable logistic regression model for AKI is shown in Table 3.

**Table 3:** Multivariable logistic regression for acute kidney injury, cluster-robust standard errors (*n* = 127, 29 events, 123 patients).

Predictor	Adjusted OR	95% CI	*p*
Catheter (yes vs. no)	0.49	0.16–1.47	0.203
Length of stay (per log-day)	3.43	1.91–6.17	<0.0001
Age (per year)	1.02	1.00–1.05	0.099

Catheter status was not significantly associated with AKI; the confidence interval was wide and crossed unity. With only 29 events for this model, this should be read as an imprecise, exploratory estimate rather than a demonstration of safety (Section 4.5).

### 3.3. Systemic Opioid Consumption

The crude narcotic-day-to-length-of-stay ratio did not differ significantly between groups (median 0.79 vs. 0.61, *p* = 0.831). Given Poisson overdispersion, we report the negative binomial model (Table 4).

**Table 4:** Negative binomial regression for systemic narcotic-days, cluster-robust standard errors (*n* = 127; offset = log length of stay; dispersion α = 0.99).

Predictor	Adjusted RR	95% CI	*p*
Catheter (yes vs. no)	1.15	0.88–1.51	0.294
Age (per year)	1.02	1.01–1.03	<0.0001

For comparison, a preliminary Poisson model with standard errors simply rescaled for overdispersion had suggested a significant association (RR 1.31, 95% CI 1.03–1.67, *p* = 0.030); this did not persist once the count model’s variance structure was correctly specified.

### 3.4. Sensitivity Analyses: ICU Days and Dose-Based (MME) Opioid Exposure

Adding ICU days as a covariate—available, directly or by verified zero-inference (Section 2.3), for 123 of 127 patients in the AKI model and 160 of 165 in the opioid-days model—left both principal findings materially unchanged (Table 5). ICU days showed no independent association with either outcome once length of stay was accounted for, consistent with ICU stay being a component of, rather than distinct from, overall length of stay.

**Table 5:** ICU-day-adjusted sensitivity models, cluster-robust standard errors.

Model	Catheter Effect	95% CI	*p*	ICU-Day Effect	*p*
AKI, logistic (*n* = 123, 26 AKI events)	OR 0.42	0.13–1.36	0.148	OR 1.01	0.76
Narcotic-days, negative binomial (*n* = 160)	RR 1.23	0.88–1.71	0.223	RR 1.00	0.70

Expressing opioid exposure as a standardized daily dose (MME/day, log-transformed) rather than a binary-day flag, as recommended in peer review, gave a concordant result: median MME/day was 17.1 in the catheter group versus 8.5 in the non-catheter group (Mann–Whitney *p* = 0.575), and in cluster-robust linear regression adjusted for age, catheter status was not significantly associated with MME/day (adjusted ratio 1.32, 95% CI 0.73–2.38, *p* = 0.361, *n* = 165; Table 6). Tramadol-family drugs, morphine, and pethidine together accounted for over 99% of narcotic-related order lines and were included in this calculation; transdermal fentanyl and oral tramadol drops (under 1% of order lines) could not be reliably converted to a discrete dose and were excluded (Section 2.3).

**Table 6:** Dose-based (MME) opioid exposure, cluster-robust log-linear regression (*n* = 165).

Predictor	Adjusted Ratio	95% CI	*p*
Catheter (yes vs. no)	1.32	0.73–2.38	0.361
Age (per year)	1.03	1.01–1.04	0.0004

### 3.5. Propensity-Score-Matched Sensitivity Analysis

The propensity-score-matched sensitivity analysis is shown in Table 7.

**Table 7:** Sensitivity analysis in the propensity-score-matched sample (26 catheter, 26 non-catheter; cluster-robust standard errors).

Outcome	Adjusted Effect	95% CI	*p*
AKI (logistic, adjusted for LOS, age)	OR 0.65	0.16–2.54	0.531
Narcotic-days (negative binomial, adjusted for age)	RR 1.18	0.83–1.68	0.350

Both outcomes remained non-significant and directionally consistent with the full-sample analyses across all six complementary analytic approaches reported here (cluster-robust regression, ICU-day-adjusted sensitivity, dose-based MME analysis, and propensity-score matching).

## 4. Discussion

### 4.1. Principal Findings

In this exploratory retrospective cohort of 181 earthquake-related hospitalizations, we did not detect a significant association between continuous peripheral regional analgesia and adjusted AKI risk or systemic opioid consumption, a pattern that persisted across cluster-robust, ICU-adjusted, dose-based (MME), and propensity-matched analyses. We want to be explicit about what this does and does not show: repeated non-significant findings do not demonstrate equivalence or safety, particularly given wide confidence intervals, a modest number of AKI events (29), incomplete clinical characterization of the study population, and the major residual confounding documented in Section 4.5. A preliminary analysis using a cruder statistical approach (Poisson with rescaled standard errors) had suggested a significant increase in opioid consumption in the catheter group; that apparent signal did not survive correct specification of the count model’s variance structure or an independent dose-based (MME) analysis, illustrating how sensitive modest-sample analyses can be to methodological choices.

### 4.2. Renal Findings in Context

The absence of a significant adjusted association between catheter use and AKI is, to our knowledge, the first cohort-level data on this question in a crush-injury population; existing evidence on regional analgesia and compartment-syndrome-related harm in limb trauma is otherwise confined to six case reports across two independent systematic reviews, with no comparative or cohort data [12,13]. We emphasize that our data do not demonstrate safety: they do not adjudicate compartment syndrome diagnoses or fasciotomy timing as a primary endpoint, could not characterize baseline renal risk or injury severity (Section 4.5), and the confidence interval around the catheter effect (OR 0.49, 95% CI 0.16–1.47) is compatible with both a meaningfully protective and a meaningfully harmful true effect. This complements, without resolving, the existing Kahramanmaraş-cohort literature on dialysis and mortality predictors in crush syndrome [2,24,25] and pathophysiological reviews of crush-related AKI [3,4].

### 4.3. Opioid Consumption, Including a Dose-Based Reassessment

Peer review correctly noted that an opioid-days flag does not reflect administered dose. Re-expressing exposure as MME per day gave a result concordant with the days-based analysis (adjusted ratio 1.32, 95% CI 0.73–2.38, *p* = 0.361): no significant difference, with a point estimate directionally above 1 in both metrics. Taken together with the broader surgical literature, in which an opioid-sparing regional anesthesia–analgesia strategy is often, though not uniformly, feasible across procedure types [5,26], we interpret this cohort’s data as not showing a clear opioid-sparing effect of catheter use, without concluding a true increase in requirement; the modest, clinically uncharacterized catheter group and the width of the confidence intervals mean a true effect in either direction cannot be excluded.

We also considered, as raised in peer review by a reviewer experienced with continuous peripheral nerve blockade, a further possible explanation: a well-functioning catheter is expected to meaningfully reduce systemic opioid requirements, and the absence of a detectable reduction here may reflect variable block efficacy rather than a true absence of effect. Our dataset contains no block-specific quality indicators—sensory level, local anesthetic infusate concentration or rate, catheter-tip location confirmation, or clinician-assessed block adequacy—so we cannot distinguish a catheter that was functioning well but insufficient for the pain burden of repeated debridement in this severely injured subgroup from one that was, in some patients, inadequately effective. This is a concrete, testable question for prospective work that captures block-quality indicators directly (Section 4.5).

### 4.4. A Safety Dimension Not Addressed Here: Catheter-Related Infection

Because catheters in this cohort were maintained over a length of stay nearly three times that of non-catheterized patients, prolonged catheter dwell time is a relevant safety consideration this dataset could not address, as catheter-site infection was not separately coded. Duration beyond 48–72 h, trauma status, and ICU admission are consistent predictors of perineural catheter infection in registry and systematic-review evidence, albeit at low absolute incidence [15,16], and a military polytrauma cohort similarly reported this as a real, if infrequent, complication of extended use [17].

### 4.5. Limitations

This study has substantial limitations that, in our assessment, cannot be fully resolved with the available dataset and should temper interpretation accordingly. First, no prospective sample-size or power calculation was performed; this is a retrospective, exploratory analysis of the complete available dataset, and the resulting AKI event count (29 overall) is modest for a multivariable model. Second, and most importantly, the study population is insufficiently characterized: comorbidities, baseline renal function, a formal injury-severity score (e.g., ISS), the number of compartments affected, amputation status, and the anatomical extent of crush injury were not available in the source institutional records for either group, and could not be added to this analysis. Third, confounding by indication [18] is a central concern, evident in the marked, unexplained imbalance in length of stay and surgical burden between groups; our propensity score could draw on only age and sex, the sole reliably available pre-treatment covariates, and length of stay remained substantially imbalanced even after matching—direct evidence that residual, unmeasured confounding by injury severity was not resolved by the methods available to us. Fourth, repeated non-significant findings across our sensitivity analyses (ICU-adjusted, dose-based, matched) should not be read as evidence of equivalence or safety, given the wide confidence intervals throughout. Fifth, the AKI definition relied on the first available in-hospital creatinine as a pragmatic reference rather than a true pre-injury baseline [22], likely underestimating AKI present at admission. Sixth, catheter type and anatomical location, compartment syndrome diagnoses, fasciotomy-specific rates, and catheter-site infection could not be extracted from available records. Seventh, we have no block-specific quality indicators (sensory level, infusate concentration/rate, catheter-tip confirmation) and so cannot determine whether the absence of an opioid-sparing effect reflects variable block efficacy rather than a true absence of effect (Section 4.3); nor do we have anesthesia type (general versus regional) recorded per individual surgical episode, which would be needed to test whether catheters reduced cumulative general-anesthetic exposure across repeated debridements, a rationale for catheter use raised in peer review (Section 1). Eighth, we report six complementary analyses (two primary models and four sensitivity analyses) for each outcome; we did not apply a formal correction for multiplicity, and their concordance should be read as a robustness check on the primary estimates—evidence that the finding is not an artifact of any single modeling choice—rather than as six independent confirmatory tests of the same hypothesis. Finally, this is a single-center, exploratory experience; its findings should be regarded as hypothesis-generating and require confirmation in better-characterized, adequately powered, ideally multicenter cohorts before informing clinical practice.

## 5. Conclusions

In this exploratory, hypothesis-generating cohort of earthquake-related crush injury, we did not detect a significant association between continuous peripheral regional analgesia and either adjusted AKI risk or systemic opioid consumption, a pattern consistent across six complementary analyses (cluster-robust regression, ICU-day adjustment, dose-based MME assessment, and propensity-score matching). We are explicit that these non-significant findings do not demonstrate equivalence or safety: they arise from a modest, clinically incompletely characterized cohort with wide confidence intervals and substantial, only partially addressed confounding by indication, most visibly a length-of-stay imbalance that persisted after matching. These results should not be used to inform clinical practice directly; they instead motivate a specifically designed, adequately powered, multicenter study that captures a formal injury-severity index, comorbidity and baseline renal data, amputation status, catheter type and location, catheter-related infectious complications, block-quality indicators, and anesthesia type (general versus regional) recorded per surgical episode.

## Figures and Tables

**Table 1:** Unadjusted comparison between catheter and non-catheter groups.

Variable	Catheter (*n*)	Catheter Median (IQR)	No Catheter (*n*)	No Catheter Median (IQR)/*p*
Age, years	34	32 (24–41)	138	28 (16–45) *p* = 0.369
Length of stay, days	37	42 (19–78)	137	16 (7–35) *p* = 0.0001
Surgical procedures, *n*	37	10 (6–13)	144	2 (1–4) *p* < 0.0001
Systemic narcotic-days	37	20 (0–50)	137	6 (0–14) *p* = 0.0020
Peak creatinine, mg/dL	28	0.78 (0.57–1.28)	112	0.64 (0.48–0.90) *p* = 0.148
Creatinine rise (peak–first), mg/dL	28	0.16 (0.07–0.22)	112	0.02 (0.00–0.14) *p* = 0.0004

## Data Availability

De-identified data are available from the corresponding author on reasonable request.

## Supplementary Materials

The following supporting information can be downloaded at: https://www.mdpi.com/article/10.3390/jcm15218263/s1, STROBE Statement—Checklist of items that should be included in reports of cohort studies.
