# Supplementary material for: Safety and Analgesic Consumption Impact of Continuous Peripheral Regional Analgesia in Earthquake-Related Crush Injury: A Retrospective Cohort Study
Source: J Clin Med. 2026 Oct 23;15(21):8263. doi: 10.3390/jcm15218263 (PMC13764122; doi:10.3390/jcm15218263)
Supplement: Supplementary file 1 [file jcm-15-08263-s001.zip › jcm-4595184-supplementary.pdf]

STROBE Statement — Checklist of items that should be included in reports of cohort studies

Manuscript: "Safety and Analgesic Consumption Impact of Continuous Peripheral Regional Analgesia in Earthquake-Related Crush Injury: A Retrospective Cohort Study." Based on von Elm E, et al. STROBE Statement. PLoS Med. 2007;4(10):e296.

Item No.	Recommendation	Reported on page / section
**Title and abstract**		
1a	Indicate the study's design with a commonly used term in the title or the abstract.	p.1, Abstract ("retrospective cohort study")
1b	Provide in the abstract an informative and balanced summary of what was done and what was found.	p.1, Abstract
**Introduction**		
2	Explain the scientific background and rationale for the investigation being reported.	p.1–2, §1 Introduction, ¶¶1–3
3	State specific objectives, including any prespecified hypotheses.	p.2, §1 Introduction, final ¶
**Methods**		
4	Present key elements of study design early in the paper.	p.2, §2.1 Design and Setting
5	Describe the setting, locations, and relevant dates, including periods of recruitment, exposure, follow-up, and data collection.	p.2, §2.1 Design and Setting
6a	Cohort study — give the eligibility criteria, and the sources and methods of selection of participants.	p.2–3, §2.2 Population
6b	Cohort study — for matched studies, give matching criteria and number of exposed and unexposed.	p.6, §3.1 (propensity-score model, Table 2) and §3.5 (matched sensitivity analysis, Table 7; 26 vs 26)
7	Clearly define all outcomes, exposures, predictors, potential confounders, and effect modifiers. Give diagnostic criteria, if applicable.	p.3, §2.3 Data Sources and Variable Definitions

8	For each variable of interest, give sources of data and details of methods of assessment. Describe comparability of assessment methods if there is more than one group.	p.3, §2.3 Data Sources and Variable Definitions
9	Describe any efforts to address potential sources of bias.	p.3–4, §2.4 (cluster-robust SE, negative binomial model, propensity-score matching)
10	Explain how the study size was arrived at.	p.2–3, §2.2 Population (all eligible hospitalizations in the institutional dataset)
11	Explain how quantitative variables were handled in the analyses. If applicable, describe which groupings were chosen and why.	p.3–4, §2.4 (age, log-transformed length of stay)
12a	Describe all statistical methods, including those used to control for confounding.	p.3–4, §2.4 Statistical Analysis
12b	Describe any methods used to examine subgroups and interactions.	Not applicable — no subgroup/interaction analyses were performed
12c	Explain how missing data were addressed.	p.4–5, §2.4 and Table footnotes (complete-case analysis; n reported at each step)
12d	Cohort study — if applicable, explain how loss to follow-up was addressed.	Not applicable — in-hospital outcomes only, no post-discharge follow-up
12e	Describe any sensitivity analyses.	p.6, §2.4 (sensitivity analyses described); results in §3.4–3.5
**Results**		
13a	Report numbers of individuals at each stage of the study.	p.5, §3 Results, ¶1; see Figure 1 (flow diagram)

13b	Give reasons for non-participation at each stage.	Figure 1 legend
13c	Consider use of a flow diagram.	Figure 1
14a	Give characteristics of study participants and information on exposures and potential confounders.	p.5, Table 1
14b	Indicate number of participants with missing data for each variable of interest.	Table 1 (n given per row); Figure 1
14c	Cohort study — summarise follow-up time.	p.3, §2.3 (length of stay used as the observation window)
15	Cohort study — report numbers of outcome events or summary measures over time.	p.6–7, Tables 3 and 4 (29 AKI events; narcotic-days)
16a	Give unadjusted and confounder-adjusted estimates and their precision. Make clear which confounders were adjusted for and why.	p.5–8, Tables 1–7
16b	Report category boundaries when continuous variables were categorized.	Not applicable — continuous variables (age, length of stay) were modeled continuously, not categorized
16c	If relevant, consider translating relative risk into absolute risk for a meaningful time period.	Not performed — discussed as a direction for future work
17	Report other analyses done — subgroup, interaction, and sensitivity analyses.	p.7–8, §3.4–3.5; Tables 5, 6 and 7 (ICU-day sensitivity, dose-based MME, and propensity-matched analyses)
**Discussion**		
18	Summarise key results with reference to study objectives.	p.8–9, §4.1 Principal Findings
19	Discuss limitations, taking into account sources of potential bias. Discuss direction and magnitude of bias.	p.10, §4.5 Limitations
20	Give a cautious overall interpretation considering objectives, limitations, multiplicity of analyses, and similar studies.	p.8–10, §4.1–4.4

21	Discuss the generalisability (external validity) of the results.	p.10, §4.5 Limitations, final sentence
**Other information**		
22	Give the source of funding and the role of funders, including for the original study on which the present article is based.	p.11, Funding; Relationship to Prior Publication

Note: page numbers refer to the manuscript as currently typeset in the authors' working draft and should be verified against the final journal proof before submission.
